# A Superior Two-Dimensional Phosphorus Flame Retardant: Few-Layer Black Phosphorus

**DOI:** 10.3390/molecules28135062

**Published:** 2023-06-28

**Authors:** Taiming Zhang, Huanyu Xie, Shuai Xie, Ajuan Hu, Jie Liu, Jian Kang, Jie Hou, Qing Hao, Hong Liu, Hengxing Ji

**Affiliations:** 1 State Key Laboratory of Digital Medical Engineering, School of Biological Science and Medical Engineering, Southeast University, 2# Sipailou, Nanjing 210096, China; zhangtm@seu.edu.cn (T.Z.);; 2 Department of Materials Science and Engineering, CAS Key Laboratory of Materials for Energy Conversion, iChEM (Collaborative Innovation Center of Chemistry for Energy Materials), CAS Center for Excellence in Nanoscience, University of Science and Technology of China, Hefei 230026, China; 3 School of Resource Environment and Safety Engineering, University of South China, Hengyang 421001, China

**Keywords:** black phosphorus, flame retardant, two-dimensional material, polymer

## Abstract

The usage of flame retardants in flammable polymers has been an effective way to protect both lives and material goods from accidental fires. Phosphorus flame retardants have the potential to be follow-on flame retardants after halogenated variants, because of their low toxicity, high efficiency and compatibility. Recently, the emerging allotrope of phosphorus, two-dimensional black phosphorus, as a flame retardant has been developed. To further understand its performance in flame-retardant efficiency among phosphorus flame retardants, in this work, we built model materials to compare the flame-retardant performances of few-layer black phosphorus, red phosphorus nanoparticles, and triphenyl phosphate as flame-retardant additives in cellulose and polyacrylonitrile. Aside from the superior flame retardancy in polyacrylonitrile, few-layer black phosphorus in cellulose showed the superior flame-retardant efficiency in self-extinguishing, ~1.8 and ~4.4 times that of red phosphorus nanoparticles and triphenyl phosphate with similar lateral size and mass load (2.5~4.8 wt%), respectively. The char layer in cellulose coated with the few-layer black phosphorus after combustion was more continuous and smoother than that with red phosphorus nanoparticles, triphenyl phosphate and blank, and the amount of residues of cellulose coated with the few-layer black phosphorus in thermogravimetric analysis were 10 wt%, 14 wt% and 14 wt% more than that with red phosphorus nanoparticles, triphenyl phosphate and blank, respectively. In addition, although exothermic reactions, the combustion enthalpy changes in the few-layer black phosphorus (−127.1 kJ mol^−1^) are one third of that of red phosphorus nanoparticles (−381.3 kJ mol^−1^). Based on a joint thermodynamic, spectroscopic, and microscopic analysis, the superior flame retardancy of the few-layer black phosphorus was attributed to superior combustion reaction suppression from the two-dimensional structure and thermal nature of the few-layer black phosphorus.

## 1. Introduction

Unpredictable fires pose serious challenges as to how such fires can be avoided and controlled in the future. Different fire management techniques have been envisaged, of which using flame retardants has been proven to be effective [1,2,3,4]. Among the diverse flame retardants currently available, phosphorus flame retardants (P-FRs) have the potential to be follow-on flame retardants after halogenated variants, due to their environmentally-friendly nature, high efficiency, and huge abundancy [2,5,6]. P-FRs are already being widely used, consisting of up to a quarter of the worldwide market in flame retardants [3,7,8,9,10,11,12]. Thereinto, red phosphorus (RP) has been used as a commercial additive in flammable polymers, including polyurethane, epoxy resins, and so on [13,14], to suppress their flammability. In addition, various organophosphorus varieties, triphenyl phosphate (TPP), resorcinol bis (diphenyl phosphate), and so on, have also been used to improve the flame retardancy in electronic device packaging materials [12,15]. As is well-known, phosphorous acts as an efficient flame-retardant element to suppress combustion reactions, by both promoting the formation of char in condensed phase, which blocks the transfer of heat and flammable gases, and the release of radicals (e.g., HPO_2_·, PO_2_·, etc.) in the gas phase which, in turn, neutralizes the free radicals (e.g., H·, CO·, etc.) participating in the combustion reactions [1,2].

Black phosphorus (BP), an emerging allotrope of elemental phosphorus with a two-dimensional (2D) structure, was rediscovered in 2014, and has since become one of the most attractive 2D materials after graphene, and has shown great promise for high-performance electronic and optoelectronic devices, energy storage, and biomedicine applications [16,17,18,19,20,21,22,23,24,25]. Due to an ultimate phosphorus content (100 wt.%), and good intrinsic thermal stability [26], few-layer BP is expected as a follow-on high-efficiency flame retardant after RP and organophosphorus. Few-layer BP can be prepared by exfoliation from bulk BP (e.g., mechanical exfoliation, sonication, intercalation, etc.) and synthesis from precursors of phosphorus (e.g., solvothermal methods, phase transformation under high pressure, chemical vapor phase transfer, etc.) [27,28].

Recently, few-layer BP has shown potential application as a flame retardant, and it has enhanced the flame retardancy, and even the mechanical strength, of the polymer composites. Ren et al. firstly reported the enhanced flame retardancy of waterborne polyurethane via mixing 0.2 wt.% few-layer BP into it, with the limiting oxygen index increasing by 2.6%, and the heat flow and peak heat release rate deceasing 34.7% and 10.3%, respectively [29]. Then, the combination of few-layer BP with other polymers, including epoxy resins [30,31,32,33], polyvinyl alcohol [34], polyethylene terephthalate [35], and so on [36,37,38,39,40], to enhance flame retardancy, and even mechanical properties [34,37,38,40,41,42,43], have been reported. Considering the environmental degradation of the few-layer BP [44,45,46], a further surface chemical modification was adopted to increase the stability and compatibility of the few-layer BP in polymers of few-layer BP [30,31,32,34,36,38,40], based on a large specific surface area and surface lone pair electron of few-layer BP [26,47]. All these works make few-layer BP potentially attractive for flame-retardant applications. However, it is generally believed that the universal high flame retardancy of few-layer BP mainly stems from its phosphorus element nature, and the personalities in flame retardancy of the few-layer BP among P-FRs are not fully studied and understood.

Herein, we have compared the flame retardancy of the few-layer BP to two common commercial P-FRs, RP and TPP, via constructing model polymer composites and introducing a self-extinguishing time for quantitative evaluation. The self-extinguishing time of the cellulose coated with the few-layer BP was ~56% and ~22% of those coated with red phosphorus nanoparticle and TPP, respectively, and similar results were found in polyacrylonitrile fiber films. A joint thermodynamic, spectroscopic, and microscopic analysis of the model polymer composites showed that the superior flame-retardant efficiency of the few-layer BP was attributed to a fast formation of a uniform and thermostable char layer in the condensed phase from 2D phosphorus oxide catalysis, aside from the proper oxidation temperature and low combustion enthalpy change in the few-layer BP. Beside the previous works demonstrating the potential application of BP in flammable polymers, the systematic demonstration of comparative advantages of FLBP among P-FRs in this work will further boost both fundamental research and the future technological application of FLBP in flame retardants.

In this work, we first show the process of preparing model polymer composites, followed by the combustion results of the model polymer composites. Then, based on a joint analysis of the model polymer composites, we discuss the sources of the superior flame retardancy of FLBP. Finally, a schematic diagram was exhibited to illustrate the sources.

## 2. Result and Discussion

To understand the flame retardancy characters of the few-layer BP (FLBP) among P-FRs, different P-FRs, including FLBP, RP, and TPP, were loaded on the combustible polymers, and combustions of these polymer composites were further analyzed. We first chose the commercial paper (Section 3), which mainly consisted of cellulose and was a common source of fires, as the substrate to demonstrate the flame-retardant characters.

**Figure 1:**
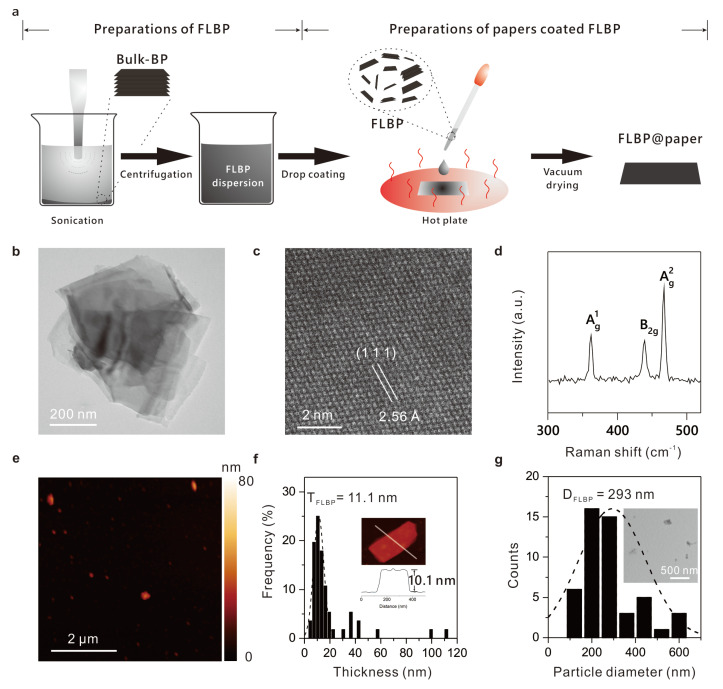
Preparation and characteristics of the few-layer BP. (**a**) The schematic of preparation of FLBP and the papers with FLBP. (**b**) A TEM image of the few-layer BP. (**c**) Atomic resolution dark field TEM image of the few-layer BP, showing the crystal structure of the few-layer BP. (**d**) A Raman spectrum of the few-layer BP. A_g_^1^, B_2g_, and A_g_^1^ are corresponding characteristic lattice vibration modes of BP. (**e**) An AFM image of the few-layer BP. (**f**) The distribution of the thickness of the few-layer BP, from analysis of the AFM images. The average thickness was ~11.1 nm. Inset: a BP flake with a thickness of 10.1 nm. (**g**) The distribution of the lateral size of the few-layer BP, from analysis of the TEM. The average lateral size was ~293 nm. Inset in (**f**): the lower magnification TEM image of the few-layer BP.

We prepared the FLBP dispersing in mixture solvents (water and alcohol (*v*:*v* = 3:1)) via a chemical vapor phase transfer method [44], followed by sonication exfoliation of bulk BP crystals (Section 3, Figure 1a) under the nitrogen environment (N_2_) to avoid BP’s degradation. A TEM (Figure 1b,c) and Raman analysis (Figure 1d) indicated that the prepared FLBP dispersed in mixture solvent with a high-quality crystal structure. With the analysis of TEM and AFM images (Figure 1e), the average thickness and lateral size of the prepared FLBP were confirmed as ~11.1 nm (Figure 1f) and ~293 nm (Figure 1g), respectively.

For comparison, RP and TPP were pre-treated. Red phosphorus nanoparticles (RPnp) with a similar lateral size to FLBP were prepared by ball milling commercial RP powders, followed by dispersion in a mixture solvent (Section 3, Figure S1), and the average lateral size of the obtained RPnp was ~204 nm (Figure S2A,B). Because TPP is soluble in the solvent, we analyzed the average lateral size of the re-precipitated TPP nanoparticles from TPP solution, which was about ~66 nm (Figure S2C). Then, the dispersions containing a certain amount of the three P-FRs were dropped on the well-cut commercial papers, followed by vacuum dry to obtain different P-FR coating paper composites (Section 3, Figure 1a and Figure S1), respectively. Additionally, the mass loadings of FLBP, RPnp, and TPP on papers (FLBP@paper, RPnp@paper, and TPP@paper) were 2.5 ± 0.7 wt%, 2.9 ± 0.6 wt%, and 4.8 ± 1.2 wt%, respectively, determined by weighing. A paper uncoated P-FRs (Paper) was wetted by the mixture solvents followed by vacuum dry to serve as the reference.

**Figure 2:**
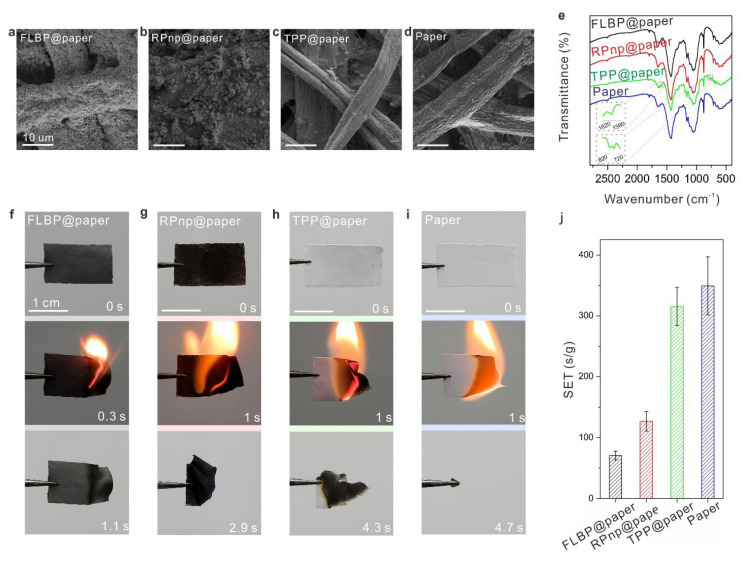
Flame-retardant performances of the papers coated with different P-FRs. SEM images of FLBP@paper (**a**), RPnp@paper (**b**), TPP@paper (**c**) and paper (**d**). (**e**) Fourier transform infrared spectra of FLBP@paper, RPnp@paper, TPP@paper and paper. The absorption features at ~1590 and ~800 − ~740 cm^−1^, assigned to C=C and P-O-C absorptions in TPP, were zoomed in the inset. Ignitability tests of FLBP@paper (**f**), RPnp@paper (**g**), TPP@paper (**h**), and paper (**i**). The flames were extinguished in ~1.1 s (**f**), ~2.9 s (**g**), ~4.3 s (**h**), and ~4.7 s (**i**), respectively. Note: the paper (**i**) burned out completely before it was extinguished. (**j**) SET values of FLBP@paper, RPnp@paper, TPP@paper, and paper. Note: the unit of SET is second per gram.

Then, the morphology and composition of the papers coated with different P-FRs were characterized. As shown in Figure 2a–d, FLBPs were uniformly distributed on the surface of paper, while RBnp on the surface of paper was partly agglomerated, due to less dispersion in solvents (Figure S3). The morphology of TPP@paper and paper was similar, indicating a uniform dispersion of TPP in cellulose. The crystal structures of the FLBP and RPnp on the papers were further confirmed by Raman spectra (Figure S4A,B). Additionally, a uniform distribution of TPP in the paper was confirmed by EDS mapping (Figure S4C).

Fourier transform infrared (FT-IR) spectra (Figure 2e) of FLBP@paper, RPnp@paper, and TPP@paper indicated that P-FRs were physically adsorbed with the papers, because there was no new absorption peak in the spectra compared to that of paper [48], except for two additional peaks (~1580–~1610 cm^−1^ and ~740–~800 cm^−1^) in the TPP@paper, attributed to C=C and P-O-C absorption in the TPP molecule [49].

Further, flame-retardant performances of the papers coated with different P-FRs were assessed in ignitability tests. They were exposed to a flame severally, the flame was removed once ignited, then the combustions of the papers were recorded. As shown in Figure 2f–i, combustions in FLBP@paper, RPnp@paper, and TPP@paper extinguished after ~1.1 s, ~2.9 s, and ~4.3 s, respectively, while paper was combusted unremittingly until full burnout. To quantitatively study the flame-retardant performance of these samples, the self-extinguishing times (SET), the combustion time divided by the corresponding mass of the whole composites [3,50], were calculated. As shown in Figure 2j, paper was highly flammable, with a SET of 349 ± 48 s g^−1^; for TPP@paper, the SET decreased a little, with the amplitude of reduction being less than 10%. The SETs values of FLBP@paper and RPnp@paper decreased to 71 ± 7 s g^−1^ (20% of paper’s SET) and 126 ± 16 s g^−1^ (36% of paper’s SET), respectively. The SET of FLBP@paper was the lowest, at a ~2.5 wt% loading of FLBP. This result showed that the flame-retardant efficiency of FLBP was the highest for cellulose among these P-FRs, as ~1.8 and ~4.4 times that of RPnp and TPP, respectively, based on comparisons in the self-extinguishing times.

**Figure 3:**
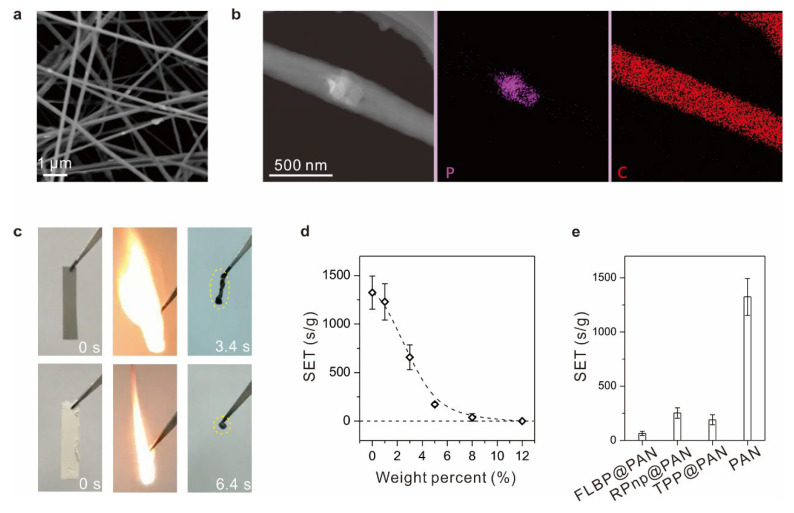
Flame-retardant performances of the PAN fibers film combining FLBP. (**a**) A SEM image of FLBP@PAN. The bright sheets were FLBP. (**b**) STEM-EDS elemental mappings of a fiber in FLBP@PAN. FLBP in the fiber. (**c**) Ignitability tests of the FLBP@PAN (up) and blank PAN fiber films. Combustion times shortened and products (marked by yellow) increased in FLBP. (**d**) The SET values of FLBP@PAN with different addition contents of FLBP. (**e**) The SET values of FLBP@PAN, RPnp@PAN, TPP@PAN, and blank PAN fiber film. The phosphorus contents in FLBP@PAN, RPnp@PAN, and TPP@PAN were similar, ~8 wt.%.

For a further illustration of the advantages of flame retardancy of FLBP, we combined FLBP with polyacrylonitrile (PAN) fiber, accounting for about the fifth in the global fiber production, and widely applicating in ultra-filtration membranes, fibers for textiles, and manufacture of carbon fiber. One of the main characteristics of PAN is its high flammability [51]. Thereafter, FLBP was compounded into PAN fiber to improve the flame-retardant properties. The PAN fiber film combining FLBP (FLBP@PAN) was prepared by electrospinning (Figure S5, Section 3). From the morphology and composition characterization results of FLBP@PAN (Figure 3a,b), FLBP was monodispersed in the PAN fibers. In the subsequent ignitability tests (Figure 3c), the PAN fiber films’ combustion time shortened, and the combustion products increased, with the addition of FLBP. Further, quantitatively assessing the flame retardancy, the SET of FLBP@PAN decreased, with an increase in the weight percent of FLBP, and the SET value decreased to zero (unignitable) when the weight percent of FLBP was ~10 wt% (Figure 3d). When compared to RPnp@PAN (PAN fiber film combining RPnp) and TPP@PAN (PAN fiber film combining TPP), the flame-retardant performance of FLBP@PAN was also superior, about 3.9 and 2.9 times that of RPnp and TPP in similar phosphorus contents load, respectively (Figure 3e).

With the presentation of the above results, the superior flame retardancy of FLBP was shown in suppressing the combustion reaction of cellulose and PAN. In other flammable polymers, FLBP could also have a good flame-retardant effect, as shown in previous research [30,34,35,38,40]. To further reveal how FLBP obtains an edge, a joint thermal, spectroscopic, and microscopic analysis of the papers coated with different P-FRs, more suitable model polymer composites than PAN fiber film in uniformity, were performed.

**Figure 4:**
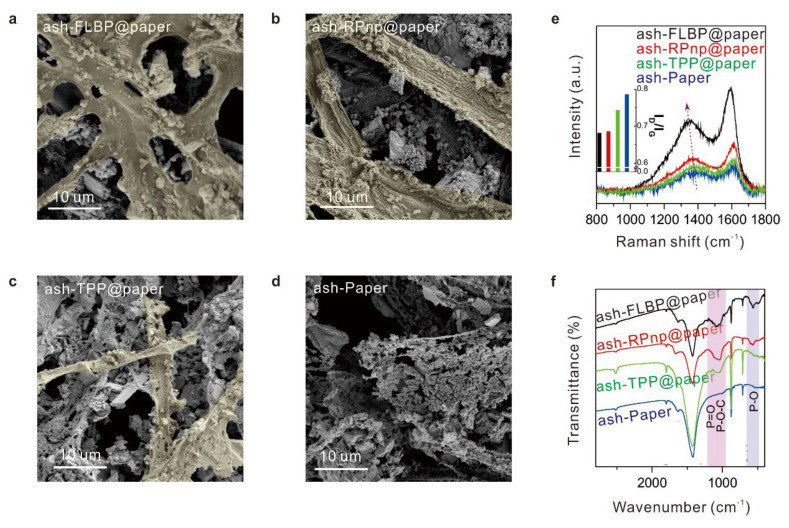
Morphology and component analysis of the combustion products of the papers with different P-FRs. SEM images of ashes of FLBP@paper (ash-FLBP@paper) (**a**), RPnp@paper (ash-RPnp@paper) (**b**), TPP@paper (ash-TPP@paper) (**c**), and blank paper (ash-Paper) (**d**). False colors have been added to mark continuous fibrous units. (**e**) Raman spectra of these four ashes. Inset: I_D_/I_G_ of these four ashes. (**f**) Fourier transform infrared spectra of these four ashes. The areas of main differences in characteristic absorption peaks were highlighted, namely P=O and P-O-C absorption peaks (mauve) and the P-O absorption peak (blue).

The combustion products of the papers coated with different P-FRs were analyzed first. The surface morphology of ashes of the papers with different P-FRs were shown in Figure 4a–d. The fibrous units of the cellulose were partly reserved in the ashes of FLBP@paper (ash-FLBP@paper), RPnp@paper (ash-RPnp@paper), and TPP@paper (ash-TPP@paper), while they decomposed into loose blocks in the ash of the reference paper (ash-Paper) (Figure 4a–d). Notably, a continuous and smooth surface (Figure 4a) was formed on the ash-FLBP@paper, while the surfaces were coarse in ash-RPnp@paper and discontinuous in TPP@paper.

Raman and FTIR spectra of these ashes were analyzed to understand their composition. As shown in the Raman spectra (Figure 4e), the graphitic (G) band and defect (D) band of ashes appeared centered at ~1590 and ~1350 cm^−1^, respectively, indicating that the ashes were made predominantly of char, consistent with previous research [31,52]. With the addition of P-FRs, the position of the D band shifted to lower wavenumbers, probably due to phosphorus doping in the chars. Under the same measurement conditions, the intensities of the D and G peaks were highest in ash-FLBP@paper, followed by ash-RPnp@paper, ash-TPP@paper, and ash-paper, indicating that the char content of the ash-FLBP@paper was the highest. Additionally, the char in FLBP@paper should have less defects in structure and higher structural stability from the Raman spectra results of lowest I_D_/I_G_ (inset in Figure 4e). [52,53,54] FT-IR spectra were investigated to further elucidate the structure of the chars. As shown in Figure 4f, it was confirmed that P-FRs were involved in the formation of chars, from the appearance of P-O-C vibration in ashes of the papers coated with P-FRs, consistent with results from Raman spectral analysis. The appearance of P-O and P=O in FTIR spectra should be attributed to the formation of phosphorus oxide resulting from oxidation of P-FRs during combustion. From these results, we deduced the formation of a more continuous, smooth, and higher quantity of the structural stable char layer in FLBP@paper during combustion.

**Figure 5:**
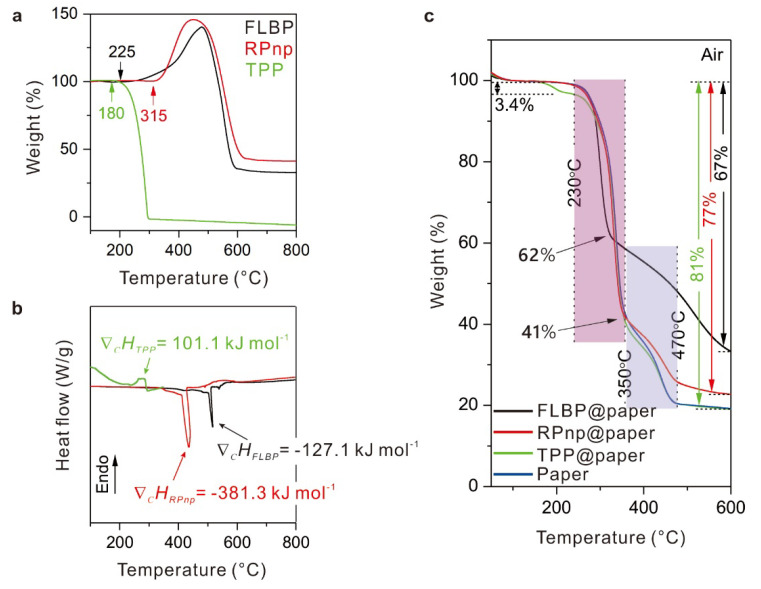
Thermal analysis of the P-FRs and the papers with P-FRs. (**a**) The TGA curves of FLBP, RPnp and TPP in air. TPP begins to volatilize at 180 °C, FLBP and RPnp begin to oxidize at 225 °C and 315 °C, respectively. The weight loss after ~450 °C in FLBP and RPnp is mainly due to volatilization of phosphorus oxides. (**b**) The DSC curve of FLBP, RPnp, and TPP in air. The combustion enthalpy changes in FLBP and RPnp were −127.1 and −381.3 kJ mol^−1^, and the volatilization enthalpy changes in TPP was 101.1 kJ mol^−1^. Note: the unit of heat flow is watts per gram. (**c**) The TGA curves of FLBP@paper, RPnp@paper, TPP@paper, and paper in air. Two main decompositions processes were marked with mauve and blue, respectively. The final weight-loss rates of different samples were noted; the weight loss of TPP@paper around 180 °C was attributed to volatilization of TPP. The similar weight gain from the oxidation of FLBP and RPnp [55,56] in (**a**) was difficult to distinguish in (**c**) because of the quick and near-half weight loss in decomposition of paper (more than 95% of the whole sample quality).

To elucidate the flame-retardant interactions between paper and P-FRs during the combustion process, the behaviors under thermal stimuli of these P-FRs, and the papers coated with these P-FRs, were analyzed by thermal analysis technologies. As shown in Figure 5a,b, although the oxidation of FLBP in air was an exothermic reaction, compared to the endothermic volatilization of TPP, the combustion enthalpy of FLBP was two times lower than that of RPnp, and the temperature of the peak of combustion enthalpy was higher than that of RPnp and TPP, indicating a superior thermal stability of FLBP.

Thermal gravimetric analysis (TGA) curves of the papers coated with P-FRs in air and N_2_ atmosphere were shown in Figure 5c and Figure S6. When heated in air, two main decomposition processes were observed. The first weight loss at around ~230–~350 °C (the mauve area in Figure 5c) results from two competing pathways: (i) the depolymerization of the glycosyl units to volatiles (partly flammable); and (ii), the decomposition of the glycosyl units to thermostable aromatic char. The subsequent weight change at ~350–~470 °C (the blue area in Figure 5c) is due to the further oxidation of char [37,38]. When in N_2_ atmosphere (Figure S6), the temperature area of the first decomposition reaction became higher, and further oxidation of char did not occur. The TGA curves of different samples showed no obvious differences under N_2_, other than obvious differences in those under air atmosphere, indicating that the oxides from P-FRs’ oxidation were the main species that affected their thermal behaviors (Figure S7), mainly in the formation of char [57,58], probably due to the dehydration of phosphoric oxide. Therefore, the low volatile temperature of TPP was unfavorable for its effect in the high temperature decomposition of polymers.

During the first decomposition process in air, the weight loss rate of FLBP@paper was the fastest in the first half of the degradation step (Figure 5c and Figure S8), because the initial oxidation temperature of FLBP was lower (225 °C, Figure 5a) and the exothermic oxidation reaction accelerated the decomposition (Figure 5b). After the first decomposition step, FLBP@paper showed a lower weight-loss rate (~21 wt%, from ~62% to ~41%, Figure 5c) than that of RPnp@paper and TPP@paper, because of the earlier formation of char restraining the following decomposition. In the second decomposition step, the rate of weight loss of FLBP@paper was minimal (Figure 5c and Figure S8), which meant that the char formed in FLBP@paper was more thermostable, consistent with the earlier Raman analysis of ash-FLBP@paper. Until thermal decomposition in air to 600 °C, the final weight-loss rates were 67%, 77%, 81%, and 81% for FLBP@paper, RPnp@paper, TPP@paper, and paper, respectively. The thermal decomposition suppression results in thermal analysis experiments were consistent with the flame-retardant performance in ignitability tests before.

**Figure 6:**
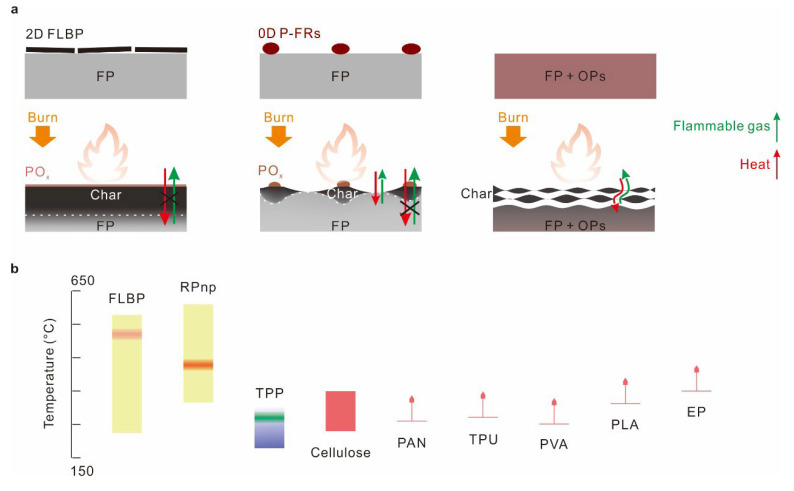
Schematic illustration of the superior flame retardancy of the few-layer BP in a structure (**a**) and a thermal-property coupling (**b**). (**a**) FP: flammable polymer, OPs: organic P-FRs. (**b**) Yellow regions represent the generation and volatilization temperature ranges of phosphorus oxide in FLBP and RPnp, and the red highlighted regions represented the regions of the combustion enthalpy peaks; the blue region represents the volatilization temperature range of TPP, where the green highlighted area represents the region of the volatilization enthalpy peak; red color in cellulose represents its decomposition to char temperature range, and different polymer decomposition start temperatures were noted. The decomposition start temperatures of polyurethane (PU), polyvinyl alcohol (PVA), polylactic acid (PLA), and epoxy resin (EP) were read from references [32,34,38,40].

With the help of morphology, component, and thermal analysis, we observed that a more continuous and thermostable char formed in the paper coated with the more thermostable FLBP, which may be one of keys to its superior flame retardancy. Considering the complications in combustion reactions and flame retardants in polymers with different thermal properties, we draw a simplified picture to intuitively demonstrate the possible advantages of FLBP in structure from the perspective of condensed phase flame retardants and in thermal-property coupling, assuming P-FRs have a similar flame-retardant effect in the gas phase [1,2,30] (Figure 6). Notably, phosphorus in FLBP was a zero-valence state, meaning free radicals with more variable valence states (e.g., PO·, PO_2_·, PO_3_·, etc.) would probably be released in the gas phase to quench the combustion reactions, rather than that in high valence-state P-FRs [1,2].

As shown in Figure 6a, 2D FLBP could be uniformly oxidized on the surface of a flammable polymer to form a continuous layer of phosphorus oxide (PO_x_), which catalyzed the fast formation of a continuous thermostable char during the decomposition of the polymer. The formed continuous char layer hindered the transmission of external heat to the interior flammable polymer, and the release of volatile fuel gases from the decomposition of flammable polymer. Thus, the combustion reaction cycle was interrupted, and the combustions were suppressed. While a continuous char layer was difficult to form quickly in the catalysis of zero-dimensional PO_x_ from the oxidation of zero-dimensional P-FRs particles, pyrocondensation, a continuous char layer was also difficult to form in a uniform distribution of organic P-FRs in flammable polymers.

From the point of view of thermal-property coupling (Figure 6b), most of the decomposition temperature ranges of different polymers were coupled with thermal stability of FLBP. Phosphorus oxides formed from FLBP could act on whole decomposition temperature ranges of these polymers to promote char formation. While the decomposition in some polymers (Cellulose, PAN, TPU, and PVA) started earlier than phosphorus oxides formed in RPnp, for TPP, it may have been volatilized before the decomposition of polymers began.

## 3. Materials and Methods

### 3.1. Preparation of the Bulk Black Phosphorus

Bulk BP was prepared through a low-pressure transport route. In detail, 500 mg of RP particle, 20 mg of Sn, and 10 mg of SnI_4_ were sealed in an evacuated quartzose ampoule. The sealed ampoule was transferred to a tube furnace and heated at 930 K for 3 h. Then, the temperature was dropped to 830 K in 15 h, followed by a natural cooling process to yield dark bulk BP. After purification, the product was stored in a glovebox for further research.

### 3.2. Preparation of the Few-Layer Black Phosphorus and Red Phosphorus Nanoparticles

FLBP was prepared by liquid exfoliation with the aid of sonication (Figure S1). In detail, the obtained bulk BP was ground and dispersed in a deoxygenated mixture solvent of water and ethanol (3:1, *v*:*v*), followed by a probe-sonic exfoliation (Scientz-IID; Ningbo Scientz Biotechnology Co., Ltd. Ningbo, China) for 3 h, at a power of 475 W. Then, the suspension was centrifuged at 6000 rpm for 10 min, and the supernatant containing FLBP was collected. FLBP powder was obtained by filtering the supernatant (filter paper pore size: 0.22 μm; diameter: 47 mm), and the residue was dried under vacuum. RPnp was prepared by ball milling the commercial RP powders in a planetary ball mill machine (PM 100, Retsch Shanghai, China). In detail, the RP powders were placed into a stainless steel ball mill capsule (100 mL) containing stainless steel balls, the capsule was sealed in a glovebox filled with Ar, then equipped on the planetary ball mill machine. The ball mill process was performed at ambient temperature for total 24 h, with a rotation speed of 200 rpm and a 30 min milling time with a 15 min interval. After the ball mill process, the capsule was cooled at room temperature, then the capsule lid was opened in the glovebox, and the RPnp was collected for further study.

### 3.3. Preparations of the Papers Coated with Different P-FRs

Papers coated with FLBP, RPnp, and TPP were fabricated by drop coating FLBP, RPnp, and TPP dispersions on commercial papers (thickness: ~100 μm. A4. TANGO Guangdong, China) (Figure S1). Taking FLBP@paper as an example, a certain amount of FLBP powder was added to the deoxygenated ethanol and water (3/1, *v*/*v*) mixture solvent, and the solution, after the ultrasonic procedure, was drop coated onto both sides of the paper, well-cut after drying in a vacuum oven at 45 °C overnight. To accelerate the evaporation of the solvent, the paper was placed on a hot plate of 80 °C and swept with nitrogen during the dropping process. After coating, the paper was placed in a vacuum oven at 45 °C overnight, and FLBP@paper was obtained. For the RPnp@paper and TPP@paper, the RPnp and TPP were dispersed and dissolved in the same deoxygenated mixture solvents, and the rest of the treatments were the same as with the FLBP@paper. The load of FLBP, RPnp, and TPP on papers were determined by weighing. In detail, we first recorded the mass of the printed paper after drying it in a vacuum oven at 45 °C overnight, then weighed the mass of the samples loaded with FLBP, RPnp, and TPP, then calculated the load. A blank paper (paper), after drop coating blank mixture solvent, was regarded as reference.

### 3.4. Preparations of the PAN Fiber Films with Different P-FRs

PAN fiber films loaded with FLBP, RPnp, and TPP were prepared by electrospinning (Figure S5). Take FLBP@PAN as an example, a certain amount of FLBP was dispersed in dimethyl formamide (DMF) by ultrasonic process, and stirred overnight, then PAN powder was added to the mixed solvent (8 wt%, PAN/DMF); after stirring overnight, a uniformly dispersed sample solution was obtained. Next, the solution was transferred to a syringe (5 mL), and the syringe was placed on an electrospinning machine; the parameters of the instrument were 20 KV (voltage), 15 cm (receive distance), and 0.1 mm min^−1^ (extrusion rate). The obtained fiber film was removed from the substrate (Al foil), followed by vacuum drying at 45 °C overnight, then the FLBP@PAN was obtained. For the RPnp@PAN and TPP@PAN, the RP nanoparticles and TPP powders were dispersed in DMF, and the rest of the process was the same as FLBP@PAN fibers. A blank PAN fiber film, with no addition of P-FRs, was regarded as reference.

### 3.5. Characterizations

Scanning electron microscopy (SEM) was performed on JSM-2100F (JEOL Ltd. Beijing, China) and the Raman spectra were acquired with a Renishaw inVia with a 532 nm laser excitation. The TEM was performed on JEM-ARM200F (JEOL Ltd. Beijing, China) at an accelerating voltage of 200 kV. The atomic force microscopy (AFM) measurements were carried out on an XE7 scanning probe microscope (Park, Korea). Thermal gravimetric analysis (TGA) was carried out on a Q600 SDT instrument (New Castle, TA, USA). Fourier transform infrared spectroscopy (FT-IR) was acquired with a Bruker Tensor 27.

## 4. Conclusions

We demonstrated the superior flame-retardant performance of FLBP among P-FRs in cellulose and PAN. More thermostable FLBPs with a 2D structure nature catalyzed the fast formation of continuous and thermostable char layers on the interface of flammable polymers, which suppressed heat and flammable gas transmission during the combustion, to quickly extinguish the flame. The flame retardancy in self-extinguishing of FLBP was found to be ~1.8 and ~4.4 times that of RPnp and TPP in cellulose, respectively, and a more continuous and smoother char layer and larger amount of char were formed after combustion in cellulose coated with FLBP than that coated with RPnp, TPP, and the blank. In addition, the combustion enthalpy changes in FLBP (−127.1 kJ mol^−1^) are one third of that of RPnp (−381.3 kJ mol^−1^) in exothermic combustion reactions. Although further research into FLBP (e.g., compatibility, stability, gas-phase flame retardant, smoke-suppression performance, and cheap preparations of FLBP, etc.) is needed, the demonstration of the comparative advantages in flame retardancy of FLBP may further boost both fundamental research and the future technological application of FLBP-base flame retardants.

## Data Availability

Data is available in the manuscript or the Supplementary Materials.

## Supplementary Materials

The following supporting information can be downloaded at: https://www.mdpi.com/article/10.3390/molecules28135062/s1, further details of the preparation, TEM and SEM measurements, Raman spectroscopy measurements, and TGA results. Figure S1: The schematic of preparation of different P-FRs and the papers with different P-FRs; Figure S2: (A) The distribution of lateral size of RPnp from analysis of TEM images. Inset in (A) is the lower magnification TEM image of RPnp. (B) Raman spectrum of the RPnp. The Raman peaks between 200 and 500 cm^−1^ were characteristic peaks of RP. (C) The distribution of lateral size of re-precipitated TPP nanoparticles from analysis of TEM images. Inset in (C) is the lower magnification TEM image of re-precipitated TPP nanoparticles; Figure S3: The photo of FLBP and RPnp dispersing in n-hexane (A), water (B) and DMF (C) after two hours of sonication with a same concentration (0.5 mg mL^−1^). N-hexane (relative polarity: 0.09), water (relative polarity: 1) and DMF (relative polarity: 0.386) were dispersants; Figure S4: (A) Ra-man spectrum of FLBP@paper. (B) Raman spectrum of RPnp@paper. (C) Energy-dispersive X-ray spectroscopy (EDS) mapping of TPP@paper; Figure S5: (A) Schematic diagram of FLBP@PAN fab-rication. (B) SEM image of FLBP@PAN. (C) Raman spectrum of FLBP@PAN, acquired from the area of bright spots in (B); Figure S6: TGA curves of the papers coated with different P-FRs under N2. The weights at 105 °C were normalized to avoid the disturbance from absorbed water, and the main decomposition process was marked with mauve. The weight loss rate of TPP@paper around 180 °C attributed to volatilization of TPP was noted; Figure S7: (A) TGA curves of FLBP@paper in air or N2. (B) SEM image of FLBP@paper after TGA test in air. The fibrous units of cellulose were partly re-served. (C) SEM image of FLBP@paper after TGA test in N2. The fibrous units of cellulose were destroyed. Indicating phosphorus oxide was the catalyst for char formation; Figure S8: DTG curves of the papers with different P-FRs under air. The peak of weight loss of FLBP@paper was at ~300 °C, and the weight-loss rate of FLBP@paper was lower than that of the others during 350~470 °C; Figure S9. TGA curve of PAN fibers in air; Table S1. BP-base flame retardants in flamable polymers.

## Sample Availability

Samples of the compounds are available from the authors.
