# Supplementary material for: A Superior Two-Dimensional Phosphorus Flame Retardant: Few-Layer Black Phosphorus
Source: Molecules. 2023 Jun 28;28(13):5062. doi: 10.3390/molecules28135062 (PMC10343849; doi:10.3390/molecules28135062)
Supplement: Supplementary file 1 [file molecules-28-05062-s001.zip › molecules-2442498-supplementary.pdf]

Supporting Information

A Superior two-dimensional phosphorus flame retardant: few-layer black phosphorus

*Taiming Zhang, Huanyu Xie, Shuai Xie, Ajuan Hu, Jie Liu, Jian Kang, Jie Hou, Qing Hao, Hong Liu, Hengxing Ji**

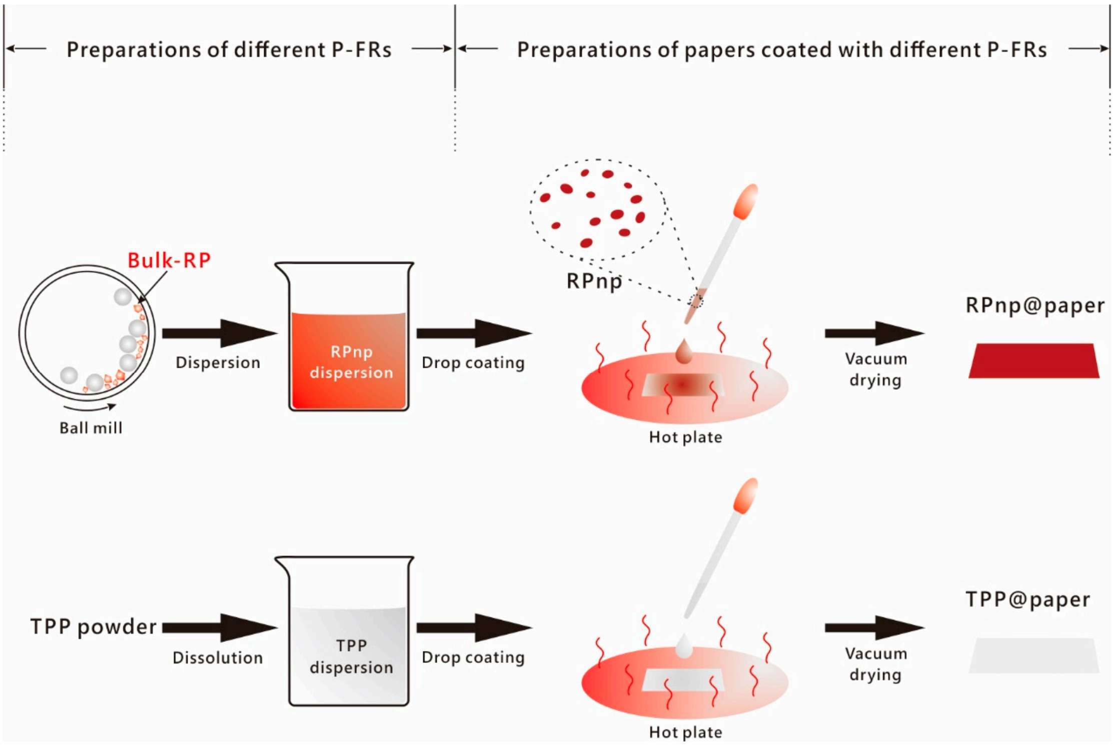

Figure S1. The schematic of preparation of different P-FRs and the papers with different P-FRs.

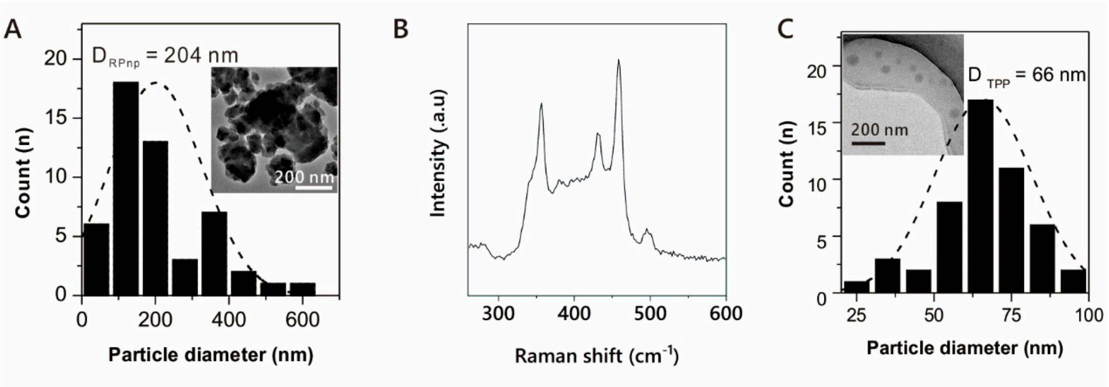

Figure S2. (**A**) The distribution of lateral size of RPnp from analysis of TEM images. Inset in (**A**) is the lower magnification TEM image of RPnp. (**B**) Raman spectrum of the RPnp. The Raman peaks between 200 and 500 cm^{-1} were characteristic peaks of RP. (**C**) The distribution of lateral size of re-precipitated TPP nanoparticles from analysis of TEM images. Inset in (**C**) is the lower magnification TEM image of re-precipitated TPP nanoparticles.

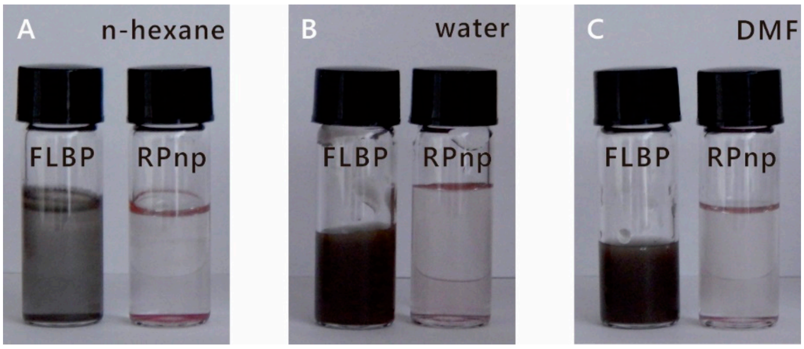

Figure S3. The photo of FLBP and RPnp dispersing in n-hexane (**A**), water (**B**) and DMF (**C**) after two hours of sonication with a same concentration (0.5 mg ml^{-1}). N-hexane (relative polarity: 0.09), water (relative polarity: 1) and DMF (relative polarity: 0.386) were dispersants.

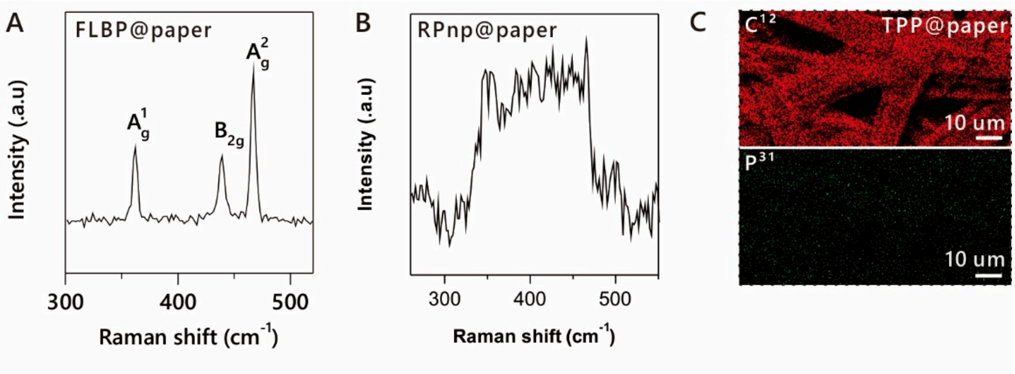

Figure S4. (**A**) Raman spectrum of FLBP@paper. (**B**) Raman spectrum of RPnp@paper. (**C**) Energy-dispersive X-ray spectroscopy (EDS) mapping of TPP@paper.

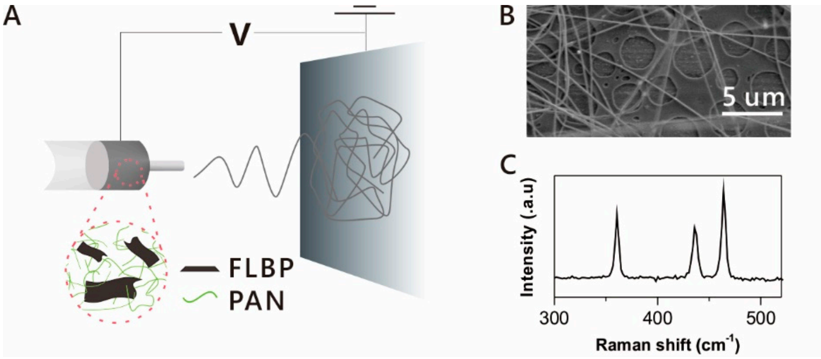

Figure S5. (**A**) Schematic diagram of FLBP@PAN fabrication. (**B**) SEM image of FLBP@PAN. (**C**) Raman spectrum of FLBP@PAN, acquired from the area of bright spots in (**B**).

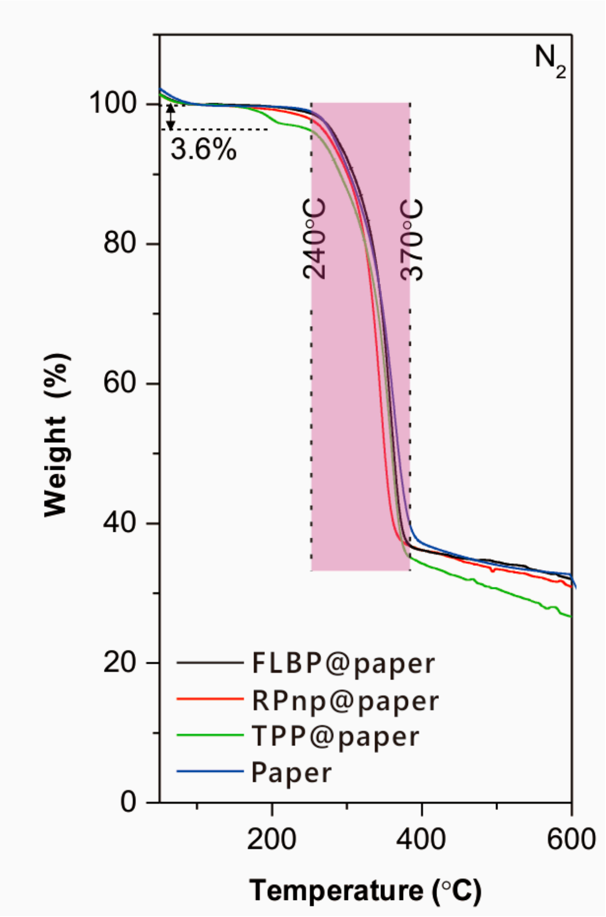

Figure S6. TGA curves of the papers coated with different P-FRs under N_2. The weights at 105℃ were normalized to avoid the disturbance from absorbed water, and the main decomposition process was marked with mauve. The weight loss rate of TPP@paper around 180℃ attributed to volatilization of TPP was noted.

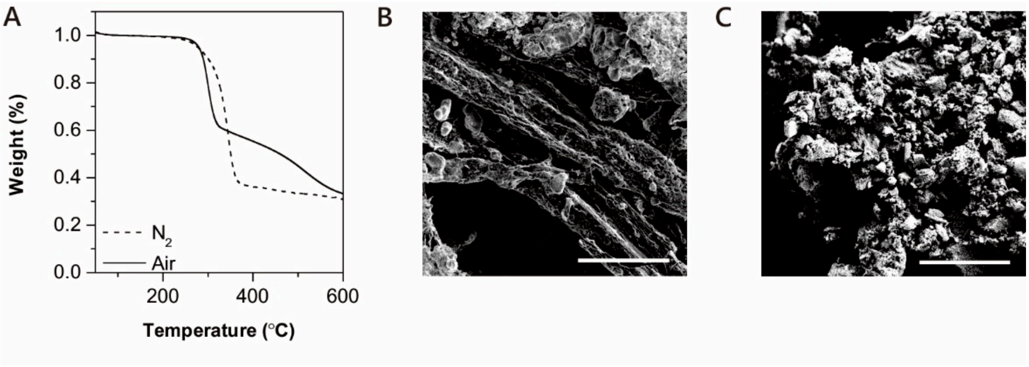

Figure S7. (**A**) TGA curves of FLBP@paper in air or N_2. (**B**) SEM image of FLBP@paper after TGA test in air. The fibrous units of cellulose were partly reserved. (**C**) SEM image of FLBP@paper after TGA test in N_2. The fibrous units of cellulose were destroyed. Indicating phosphorus oxide was the catalyst for char formation.

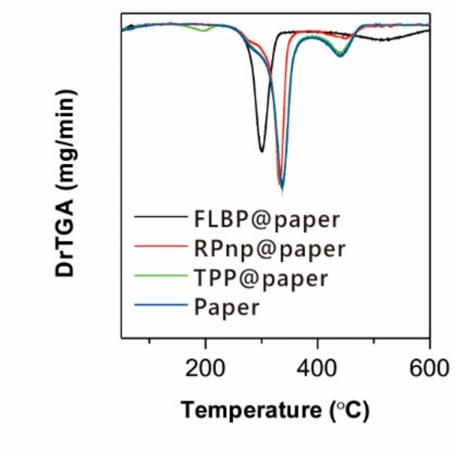

Figure S8. DTG curves of the papers with different P-FRs under air. The peak of weight loss of FLBP@paper was at ~300℃, and the weight-loss rate of FLBP@paper was lower than that of the others during 350 ~ 470℃.

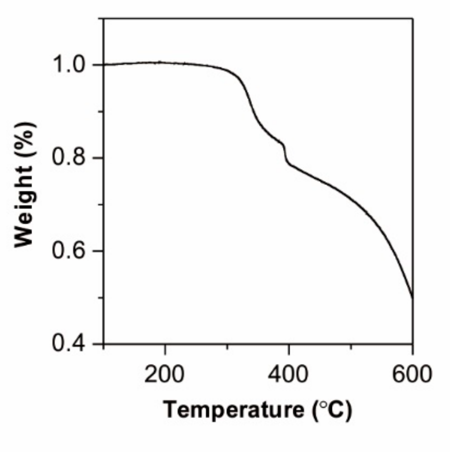

Figure S9. TGA curve of PAN fibers in air.

Table S1. BP-base flame retardants in flamable polymers

**Flame retardants**	**Polymers**	**Additive amount**	**Flame-retardant efficiency**	**References**
Black phosphorene	waterborne polyurethane	0.2 wt %	limiting oxygen index increased by 2.6%; heat flow decreased by 34.7%; peak heat release rate decreased by 10.3%	Polymers 2018, 10 (3), 227 [29]
BP-EC-Exf	polyurethane acrylate	0.1 wt %	tensile strength increased by 59.8%; tensile fracture strain increased by 88.1%; peak heat release rate reduced by 44.5%; total heat release decreased by 34.5%	ACS Applied Materials & Interfaces 2019, 11 (14), 13652-13664 [36]
BP-PZN	Epoxy Resin	2 wt%	59.4% decrease in peak heat release rate; 63.6% reduction in total heat release	Small 2019, 15 (10), 1805175 [30]
BP/G	waterborne polyurethane	3.55%	improves residues in TG analysis (5.64%) and cone calorimeter test (12.50%); a lower peak release rateand total heat release, which decrease by 48.18% and 38.63%	Polymers 2019, 11 (2), 193 [37]
BP@MF	epoxy resin	1.2 wt%	char yield increased by 70.9%; limiting oxygen index increases by 25.9%; peak of heat release rate reduced by 43.3%; fire growth rate decreases by 41.2%	Chemical Engineering Journal 2020, 382, 122991 [31]
BP-MCNTs	epoxy resin	2 wt%	peak of heat release rate and total heat release reduced by 55.81% and 41.17%; FGI decreased by 10.38	Journal of Hazardous Materials 2020, 383, 121069 [32]
BP-NH-TOF	epoxy resin	2 wt%	a decrease in PHRR (61.2%) and THR (44.3%); improved LOI (29.0%) and UL-94 (V-0)	Chemical Engineering Journal 2020, 402, 126212 [34]
TA-BP	polyurethane	2.0 wt%	peak value of heat release rate (−56.5 %), total heat release (−43.0 %), CO_2 concentration (−57.3 %)	Journal of Hazardous Materials 2020, 387, 121971 [38]

BP/BN	Waterborne Polyurethane	0.4 wt%	limiting oxygen index to 33.8%; Peak heat release rate and total heat release reduced by 50.94% and 23.92%	Polymers 2020, 12 (7), 1487 [39]
BP-CTAB	Polylactic acid	2 wt%	peak heat release rate decreases by 38.8%; the time to peak heat release rate postponed from 157 to 200 s; better than red phosphorus	Polymer Degradation and Stability 2020, 178, 109194 [40]
IL-BP	polyurethane	2 wt%	decreases of 38.2% and 19.7% in peak values of heat release rate and total heat release; the maximum concentration of CO_2 and highly toxic CO decreased by 36.9% and 26.5%	Journal of Colloid and Interface Science 2020, 561, 32-45 [41]
BP nanosheets	nanosheets	24 wt%	26.8% reduction in pHRR; 23.2% reduction in THR; 5% increment in the LOI value	Journal of Materials Chemistry A 2020, 8 (28), 14126-14134 [42]
BPs	Glass fiber reinforced polyethylene terephthalate	2.7 wt%	PHRR and THR rate decreases ca. 52.5% and 34.8%; better than red phosphorus	Polymers for Advanced Technologies 2021, 32 (9), 3515-3522 [35]
BP-NH2	graphene oxide	20 wt %	heat release rate value a 96.57% decrease	Chemistry of Materials 2021, 33 (9), 3228-3240 [43]
BP nanosheets	epoxy resin	1.0 wt%	heat release peak rate by 34.4%; total heat release by 27.0%, peak of smoke production rate by 69.2%; total production of carbon monoxide by 50.8%	Nanoscale 2022, 14 (7), 2599-2604 [33]
